# The Interpretation of the Role of a Polyketide Synthase *ClPKS18* in the Pathogenicity of *Curvularia lunata*

**DOI:** 10.3389/fmicb.2022.853140

**Published:** 2022-05-24

**Authors:** Zhixiang Lu, Shaoqing Wang, Kai Dou, Jianhong Ren, Jie Chen

**Affiliations:** ^1^School of Agriculture and Biology, Shanghai Jiao Tong University, Shanghai, China; ^2^State Key Laboratory of Microbial Metabolism, Shanghai Jiao Tong University, Shanghai, China; ^3^Suzhou PANOMIX Biomedical Tech Co., Ltd., Suzhou, China; ^4^Ministry of Agriculture Key Laboratory of Urban Agriculture (South), Shanghai Jiao Tong University, Shanghai, China

**Keywords:** *Curvularia lunata*, polyketide synthase, pathogenicity, melanization, toxin

## Abstract

Plant pathogenic fungus *Curvularia lunata* (Wakker) Boedijn causes leaf spot diseases in several plants such as *Oryza sativa*, *Sorghum bicolor* (L.) Moench, and *Capsicum frutescens.* It has been spread worldwide, specifically in maize-growing regions. The polyketide synthase (PKS) plays a significant role in secondary metabolite production and its effect on virulence. The *Clpks18* of *C. lunata* strongly correlated with its pathogenicity. The role of *Clpks18* gene on the pathogenic activity of *C. lunata* remains unclear. Hence, in this study, we analyzed the importance of *Clpks18* gene on the hyphae and conidial melanization and on the sporulation and hyphal growth. The deletion of *Clpks18* gene reduced the production of methyl 5-(hydroxymethyl)furan-2-carboxylate toxin. The virulence of Δ*Clpks18* mutant was significantly reduced compared with the wild type. The metabolomics data revealed that (R)-(-)-mellein was a vital factor in the virulence of *C. lunata*. The (R)-(-)-mellein and the toxin produced by *C. lunata* were detected in the maize leaves during its infestation. In addition, the metabolomic analysis showed that the *Clpks18* gene influences glycerolipid, non-ribosomal peptide biosynthesis, and its metabolism. This study demonstrates that the *Clpks18* gene is important for the pathogenicity of *C. lunata* by influencing the complex metabolic network.

## Introduction

Plant pathogenic fungus *Curvularia lunata* (Wakker) Boedijn causes leaf spot disease in several plants such as *Oryza sativa* (Majeed et al., 2015), *Sorghum bicolor* (L.) Moench (Liu et al., 2015), and *Capsicum frutescens* (Pei et al., 2017), and it is globally spread in the maize (*Zea mays*) growing regions (Gao et al., 2014). This pathogen can colonize. Studies on virulent factors of *C. lunata* are required to reveal its pathogenic mechanisms. The melanin (Liu et al., 2011) and toxin (Liu et al., 2009) have been identified as the important virulent factors of *C. lunata*. Melanin accumulated in the cell wall of appressorium is required to maintain the cell turgor pressure, and it is required for septin-mediated infection of plants leaves (Ryder et al., 2019). A non-host-specific toxin, methyl 5-(hydroxymethyl)furan-2-carboxylate (M5HF2C) has been successfully identified in the maize leaves infected by *C. lunata*. M5HF2C obtained from the fermentation medium of *C. lunata* showed virulence in the plants such as *O. sativa*, *Capsicum annuum*, and *S. bicolor* (Liu et al., 2009).

The previous studies showed that the polyketide synthase (PKS) plays a significant role in secondary metabolism and virulence of *Cochliobolus* species and *Setosphaeria turcica* (Condon et al., 2013). The PKS of *Bipolaris maydis* (*Cochliobolus heterostrophus*) converts malonyl-CoA to 1,3,6,8-tetrahydroxynaphthalene (1,3,6,8-THN) through the DHN-melanin synthesis pathway (Eliahu et al., 2007). The *Clpks18* gene of *C. lunata* (accession number MF114294) contains 6,571 base pairs (bp) with 6,276 bp open reading frame encoding 2,091 amino acids. The *Clpks18* gene of *C. lunata* is highly homologous with *B. maydis* BmPKS (accession number AY495659) and *S. turcica* StPKS (accession number AEE68981). The *Clpks18* deletion mutant (Δ*Clpks18*) of *C. lunata* showed albino mycelia and conidia, which supports the claim that *Clpks18* is responsible for DHN-melanin production (Gao and Chen, 2017). Meanwhile, the production of M5HF2C toxin in Δ*Clpks18* was significantly reduced than the wild type (WT). Similarly, the virulence of Δ*Clpks18* on maize leaves is also significantly lower than the WT (Gao and Chen, 2017). Our previous study revealed that the *Clpks18* gene is a key gene responsible for DHN-melanin biosynthesis and toxin production. In order to interpret the role of *Clpks18* gene in the pathogenicity against *C. lunata*, the pathways related to pathogenicity will be determined. Consequently, metabolome differences between Δ*Clpks18*, WT and OE-*Clpks18* were accomplished.

## Materials and Methods

### Fungal Strains, Plant Cultivar, Vector, and Bacterial Strains

*Curvularia lunata* WT strain CX-3, whose genome sequence is available, was used in this study (Gao et al., 2014). The *Clpks18* deletion mutant (Δ*Clpks18*) was offered by the Ministry of Agriculture Key Laboratory of Urban Agriculture (South) (MAKLUAS), Shanghai Jiao Tong University. *Zea mays* cultivars (ZHENGDAN-958) were used for virulence assays. The pCAMBIA1300 vector that contains the G418 resistance cassette is offered by MAKLUAS. This vector was used for complementation and overexpression of *Clpks18* gene. *Escherichia coli* strain DH-5α was used to construct the vectors. *Agrobacterium tumefaciens* AGL-1 was used for transformation.

### *Clpks18* Gene Complementation and Overexpression

The pCAMBIA1300 vector that contains the G418 resistance cassette comprising the G418 resistance gene under the control of *TrpC* promoter and terminator from *Aspergillus nidulans* was used for gene overexpression. The full-length sequence of *Clpks18* flanking with the promoter and terminator of *TrpC* was inserted into the *Hin*dIII-EcolI sites of pCAMBIA1300 to create plasmid pCAMBIA1300-C *Clpks18* using an infusion kit (In-Fusion HD Cloning kits, Takara, Code: 639648). The plasmid carrying both the *Clpks18* and the G418 resistance cassette was used to transform into the Δ*Clpks18* to generate the *Clpks18*-overexpression strain using the *Agrobacterium tumefaciens*-mediated transformation. The *Clpks18* gene-complementation mutants were constructed as mentioned above, except changing the *TrpC* promoter to the promoter of *C. lunata* itself. The integration at the target sites and the complementation of the *Clpks18* mutant were confirmed using the PCR. The sequences of primers for gene disruption, complementation, and PCR confirmation are shown in Supplementary Table 1. The overexpression of the *Clpks18* mutants was obtained by the methods described above, except the plasmid *Clpks18* flanking with *TrpC* promoter and the *TrpC* terminator.

### Analysis of the Sporulation and the Melanin Production in Mycelia and Conidia

Each strain was cultured in potato dextrose broth at 180 rpm at 28°C for 3–10 days. The growth rate of each strain was observed during 3–10 days. The conidia were harvested using sterile water to count on the 10th day. Each experiment was replicated three times.

### Virulence Assays

The leaves of the maize ZHENGDAN-958 seedlings at the five-leaf stage were inoculated by smearing with conidial suspensions (1.0 × 10^6^ conidia/ml) with 0.05% Tween-20 (each leaf was smeared by 100 μl conidial suspensions). The maize seeds inoculated with *C. lunata* were grown in the greenhouse with 85–95% air humidity for 2 days. The inoculated leaves were collected on 1st, 3rd, 5th, and 7th day to observe the infection. The lesions area covered per unit area was calculated. Then, the maize leaves were decolored, and the hyphae were dyed for observation and calculation. At 24 hpi, the single conidium expansion area was calculated. At 120 hpi, the hyphae expansion area per unit area was calculated (240 μm × 182 μm, 43,680 μm^2^). All those tests were performed in triplicates.

M5HF2C toxin and (R)-(-)-mellein virulence assays were done as mentioned above. The five-leaf stage maize leaves inoculated by Δ*Clpks18* strain conidial suspension with M5HF2C toxin and (R)-(-)-mellein at different concentrations (M5HF2C toxin: 60–240 ng/100 μl; (R)-(-)-mellein: 80–320 ng/100 μl). At 24 hpi, the membrane injury of the maize leaves was measured by relative conductivity.

### Observation on Infection Process

The infected leaves were collected at different time points. Then, they were sectioned, fixed, and stained for microscopic observation. The samples were observed using an optical microscope (Leica DM2500 Microscope and Leica DM6000 Microscope, Germany) and 120 kV Biology transmission electron microscope (Thermo Fisher Scientific, Tecnai G2 SpiritBiotwin, Czech).

For microscopic observation, inoculated leaves were immersed in 5 ml of alcoholic lactophenol [1 volume of phenol: glycerol: lactic acid: water (1:1:1:1) and 2 volumes of ethanol]. The leaves were evacuated for 15 min and then incubated at 65°C until the disappearance of chlorophyll pigment. The leaves were stained with 0.5% toluidine blue and decolorized using saturated chloral hydrate solution for an additional 2–24 h. Finally, the samples were stored in 50% glycerol for an optical microscope (Leica DM2500 Microscope). For transmission electron microscopy, the leaves were fixed with 2.5% glutaraldehyde in PBS (50 mM, pH 7.2) under vacuum infiltration until all the leaves sank to the bottom of the tube and stored at 4°C overnight. The tissues were then washed with 50 mM PBS and fixed in 1% osmic acid in PBS (50 mM, pH 7.2) at 4°C for 2 h. After three washes with 50 mM PBS, the tissues were dehydrated in an ethanol series (60–100%) and embedded in resin for 48 h. After finishing embedding, the samples were sectioned using Ultramicrotome (Leica EM UC7, Germany). Some samples were stained with toluidine blue for optical microscope (Leica DM6000 Microscope); the other samples were stained with uranyl acetate for 5 min and lead citrate for 5 min; and the grids were observed under the transmission electron microscope (Thermo Fisher Scientific, Tecnai G2 SpiritBiotwin) at 120 kV.

### Real-Time Quantitative PCR Assay

Each mutant and WT strain was cultured in PDB for 3 days and in Fries 3 media for 15 days. Total RNA of these strains was extracted using the TRIzol reagent (TRIzol™, Invitrogen, Code: 15596026, Unites States). The cDNA was synthesized using 1 mg RNA using the PrimeScript RT Reagent kit with gDNA Eraser (code: DRR047A; Takara, Japan). Real-time quantitative PCR (RT-qPCR) was carried out using a Hieff^®^ qPCR SYBR Green Master Mix (No Rox) kit (code:11201ES08; YEASEN, China). The PCR condition was described as mentioned in the kit above using a Light Cycler 96 Real-Time PCR System (Roch, United States). Glyceraldehyde-3-phosphate dehydrogenase (GAPDH) gene of *C. lunata* was used as the reference internal control for analyzing the relative expression levels using 2^–ΔΔ*CT*^ method. The qPCR primers used in this study are shown in Supplementary Table 1. Each experiment was carried out in triplicates.

### Metabolome Analysis of Wild Type and Mutants

Wild-type and mutant strains were cultured in Fries 3 media (Supplementary Table 4) at 28°C, 180 rpm, for 15 days to extract metabolite. The extracellular metabolites were extracted using the fermented culture filtrates. The intracellular metabolites were extracted from cells homogenized with liquid nitrogen pestle. The method of metabolic extraction was shown in Supplementary Information (Smart et al., 2010). The extracted metabolites were analyzed using LC-MS (Sangster et al., 2006; Want et al., 2010). Chromatographic separation of the extracted metabolites was accomplished in an Acquity UPLC system equipped with an ACQUITY UPLC HSS T3 (150 × 2.1 mm, 1.8 μm, Waters) column maintained at 40°C. The temperature of the autosampler was 4°C. Gradient elution of analytes was carried out with 0.1% formic acid in water (A) and 0.1% formic acid in acetonitrile (B) at a flow rate of 0.25 ml/min; 10 μl of each sample was injected after equilibration. An increasing linear gradient of solvent B (v/v) was used as follows: 0∼1 min, 2% B, 1–9.5 min, 2–50% B, 9.5–14 min, 50–98% B, 14–15 min, 98% B, 15–15.5 min, 98–2% B, 15.5–17 min, 2%. The ESI-MSn experiments were carried out using Thermo LTQ-Orbitrap XL mass spectrometer with a spray voltage of 4.8 and −4.5 kV in positive and negative modes, respectively. Sheath gas and auxiliary gas were set at 45 and 15 arbitrary units, respectively. The capillary temperature was maintained at 325°C. The voltages of capillary and tube were 35 and 50 V, and −15 and −50 V in the positive and negative modes, respectively. The Orbitrap analyzer scanned over a mass range of 89–1,000 m/z for the full scan at a mass resolution of 60,000. Data-dependent acquisition (DDA) MS/MS experiments were performed with a CID scan. The normalized collision energy was set at 30 eV. Dynamic exclusion was implemented with a repeat count of 2 and an exclusion duration of 15 s.

The aggregation level clustering was used in this experiment. Before the multivariate statistical analysis, the data were processed by mean-centering and scaled to unit variance conversion. Multivariate statistical analyses, such as principal component analysis (PCA), partial least squares-discriminant analysis (PLS-DA), and orthogonal partial least squares discriminant analysis (OPLS-DA), were used to analyze the results.

### Analysis of M5HF2C Toxin Production and (R)-(-)-Mellein Secreted Into Maize Leaves

The mutants and the WT strains were cultured in Fries 3 medium for 15 days under the same conditions described in the metabolomic analysis to check the M5HF2C toxin production. The M5HF2C toxin and (R)-(-)-mellein in the maize leaves were extracted using methanol, H_2_O, and formic acid (70:29:1) solution; dried using a vacuum rotary evaporator; and redissolved in methanol for UPLC-3Q-MS analysis. The extracts were analyzed using an ultra-high-performance liquid chromatography triple quadrupole mass spectrometer system (UHPLC 30A/Sciex Quadrupole 5500, AB Sciex) to determine the quantity of M5HF2C.

## Results

### Influence of *Clpks18* on the Growth and Development of *Curvularia lunata*

**FIGURE 1:**
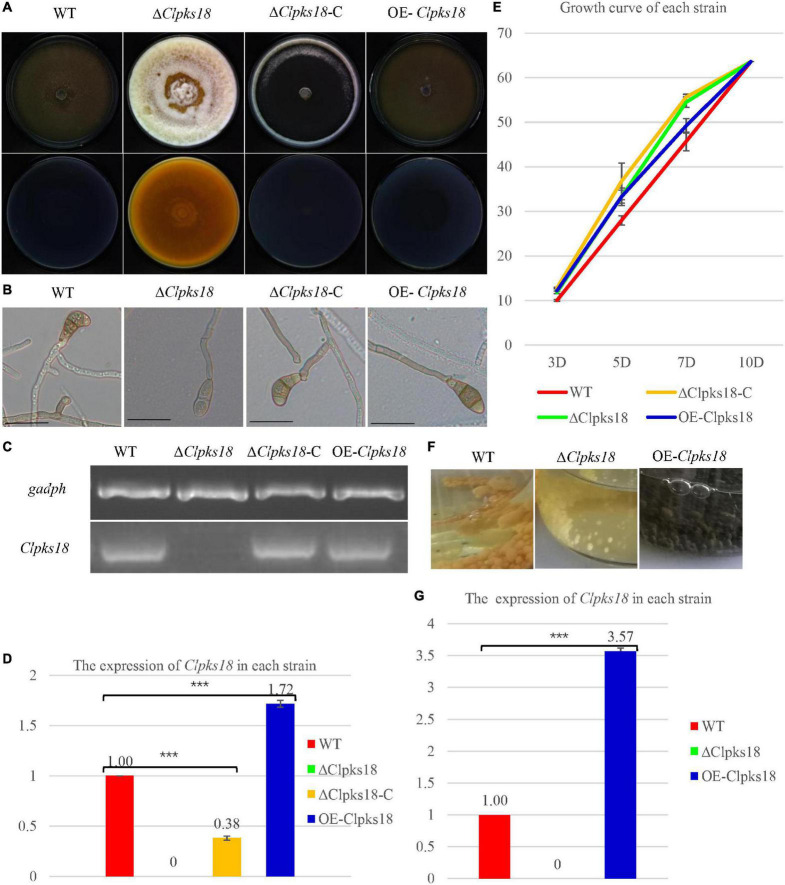
*Clpksl8* is involved to the growth and development of *Curvularia lunata*. **(A)** Cultures of *Curvularia lunata* WT, the Δ*Clpksl8* mutant, *Clpksl8*-C strain and OE-*Clpksl8* mutant grown on PDA medium for 10 days at 28°C. **(B)** *C. lunata* WT, the Δ*Clpksl8* mutant, Δ*Clpksl8*-C strain and OE-*Clpksl8* mutant grown on PDA medium for 7 days at 28°C. The scale bars represent 50 μm. **(C)** Expression levels of *Clpksl8* in the mutants and wild-type strain (WT) detected by reverse transcription-polymerase chain reaction (RT-PCR). Glyceraldehyde-3-phosphate dehydrogenase (*gadph*) was used as internal reference. **(D)** Differential expression of *Clpksl8* in different mutants compared to WT at 7 days on the PDA medium by RNA-seq analysis. ^***^ Indicates statistical significance (*p* < 0.001) using Tukey’s multiple comparison test. **(E)** The area of each strain grown on PDA medium from the 3rd day to the 10th day. Data are means ± Standard Deviation (SD) (*n* = 3), area unit: cm^2^. **(F)** Growth of the mutants and WT strains in Fries 3 medium for 15 days. Note the melanization of mycelia of OE-*Clpksl8* mutant compared with WT and Δ*Clpksl8* mutant. **(G)** Differential expression of *Clpksl8* in different mutants compared to WT in Fries 3 medium at the 7th day by RNA-seq analysis. Bars represent the mean values ± SD. *** indicates statistical significance (Two-tailed *P*-value < 0.001) using *t*-test.

*Clpks18* gene is essential for the melanization of hyphae and conidia in *C. lunata*. The Δ*Clpks18* strain resulted in the albino mycelia and conidia. The DHN-melanin production was recovered in the *Clpks18* complementation strain (Figures 1A,B). The melanization was increased in the *Clpks18* overexpression strain (Supplementary Figure 1). The expression of *Clpks18* gene in the Δ*Clpks18*-C strain was lower than the WT in the PDA medium (Figures 1C,D). Similarly, the melanization of mycelia and conidia was lower than the WT (Supplementary Figure 1). The melanization was positively correlated with the expression of the *Clpks18* gene. The Δ*Clpks18* and Δ*Clpks18*-C strains produced dense aerial mycelia compared with the WT and OE-*Clpks18* strains. The aerial mycelia of WT and OE-*Clpks18* strain were reduced during conidial production (Figure 1A and Supplementary Figure 1). The production of conidia in Δ*Clpks18* strain was reduced compared with the WT. The conidia of Δ*Clpks18*-C strain were also reduced compared with the WT. The overexpression of *Clpks18* increased the production of conidia (Table 1). However, the growth of Δ*Clpks18* and Δ*Clpks18*-C was faster than that of WT and OE-*Clpks18* (Figure 1E). It revealed that the lower expression of *Clpks18* induces the *C. lunata* to grow faster and produce the aerial hyphae. On the contrary, the highest expression of *Clpks18* induces the *C. lunata* to produce more conidia. In Fries 3 medium, the expression of *Clpks18* in the WT strain was reduced compared with the OE-*Clpks18* strain (Figure 1G). So, it revealed that the hyphal melanization of the WT strain was slower than that of the OE-*Clpks18* strain (Figure 1F).

**TABLE 1:** Conidial quantity of each strain.

	Conidial quantity	*SD*
WT	5.52 × 10^6^A	±7.31 × 10^5^
Δ*Clpks18*	1.17 × 10^4^C	±1.36 × 10^3^
Δ*Clpks18*-C	3.50 × 10^5^B	±1.99 × 10^5^
OE-*Clpks18*	5.61 × 10^6^A	±2.80 × 10^5^

*Conidial quantity of each strain grown on PDA medium was counted at the 10th day. Data are means ± SD (n = 3), unit: conidial quantity/ml. Statistically significant analysis of variance was analyzed using least significant difference tests. Different letters in each data column indicate significant differences at P = 0.01.*

### *Clpks18* Is Necessary for the Virulence of *Curvularia lunata*

**FIGURE 2:**
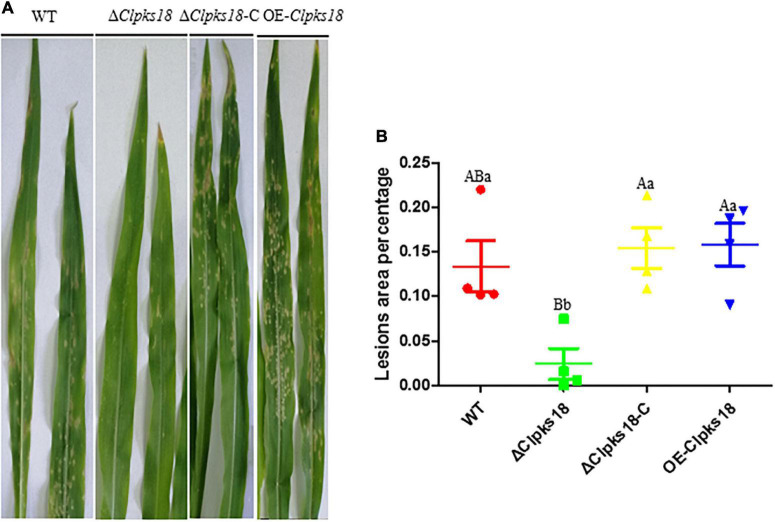
Virulence assays of the WT and mutants in maize. **(A)** The bioassay of mutants and WT in maize. The leaves of ZHENGDAN-958 at five-leaf stage was inoculated with a 0.05% Tween-20 suspension of 1 10^6^conidia per ml for 7 days. **(B)** Dot plot showing the percentage of disease lesions observed in each individual leaf harvested (*n* = 4). The capital Indicates statistical significance (*p* < 0.01) and the lowercase indicates statistical significance (*p* < 0.05) using Tukey’s multiple comparison test.

**FIGURE 3:**
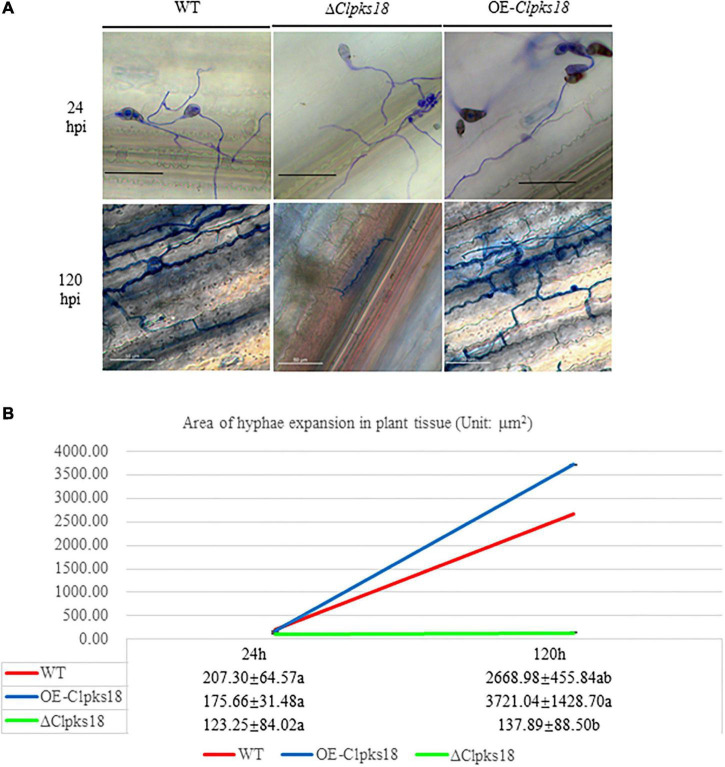
The infection process of WT strain, Δ*Clpksl8* mutant and OE-*Clpksl8* mutant of *C. lunata*. **(A)** The conidia germination of WT, Δ*Clpksl8*, OE-*Clpal8*, respectively, on the maize leaves at 24 hpi. The scale bars represent 25 μm. The infection of WT, Δ*Clpksl8*, OE-*Clpal8*, respectively, on the maize leaves at 120 hpi. The scale bars represent 50 μm. **(B)**. The area of hyphae expansion in maize leaves tissue. Data are means ± Standard Deviation (SD). Different letters denote significant differences based on Tukey’s multiple Comparison Test (*P* < 0.05).

Deletion mutant of *Clpks18* reduces virulence to maize compared with the WT and OE-*Clpks18* strains. The lesions area of the maize leaves inoculated with WT and OE-*Clpks18* strains at 7 days post-inoculation (dpi) was higher than the leaves inoculated with Δ*Clpks18* strains, which demonstrates that *Clpks18* was important to the virulence (Figures 2A,B). The conidial germination on the surface of the maize leaves was observed at 24 h post-inoculation (hpi) to validate the role of *Clpks18* gene on the infection. The results showed that the conidial germination was normal in all strains. The main differences observed between different strains were the accumulation of DHN-melanin in OE-*Clpks18* and WT strains, which was higher than that in the Δ*Clpks18* strains. However, the hyphae could directly penetrate the cuticle on the leaf surface and grow in the intercellular spaces of the leaf cells (Figure 3A). The hyphae extended between the intercellular spaces of leaves on 5 dpi (120 hpi) (Figures 3A,B). The leaves inoculated with Δ*Clpks18* strain showed the presence of hyphae in the intercellular spaces of cells without much marked lesions. However, the quantity of hyphae is much lesser than the WT and OE-*Clpks18* strains. Meanwhile, the hyphae of Δ*Clpks18* strain are slenderer than the WT and OE-*Clpks18* strains (Figure 3B and Supplementary Figure 2). The results revealed that the *Clpks18* gene was important for the pathogenicity of *C. lunata*.

### Impact of *Clpks18* on the Secondary Metabolism of *Curvularia lunata*

**FIGURE 4:**
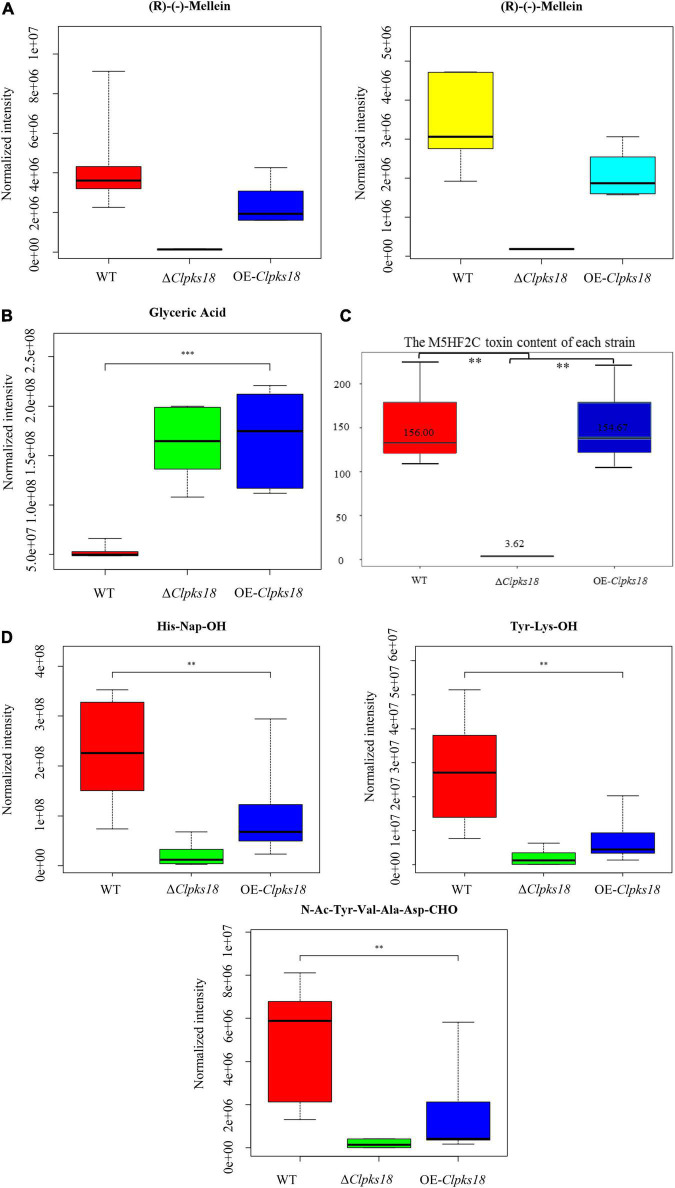
The secondary metabolites which are related to *Clpks18* gene. **(A)** The relative content of (R)-(-)-Mellein in the WT cells (Yellow), the Δ*Clpksl8* strain cells (Middle) and the OE-*Clpks18* strain cells (Turquoise); The relative content of (R)-(-)-Mellein in the WT (Red) secretions, the Δ*Clpksl8* strain secretions (Middle) and the OE-*Clpks18* strain secretions (Blue). **(B)** The relative content of Glyceric Acid in the WT secretions, the Δ*Clpksl8* strain secretions and the OE-*Clpks18* strain secretions. The error bars represent standard deviation. **(C)** Data are presented as the average M5HF2C toxin concentration in Fries 3 media at the 15th day and the bars represent the median ± maximum and minimum. ** Indicates statistical significance (*p* < 0.01) using Tukey’s multiple comparison test. *** Indicates statistical significance (*p* < 0.001) using Tukey’s multiple comparison test. Content unit (ng/mL). **(D)** The relative content of the short peptides in the WT secretions, the Δ*Clpksl8* strain secretions and the OE-*Clpksl8* strain secretions. The error bars represent standard deviation.

**FIGURE 5:**
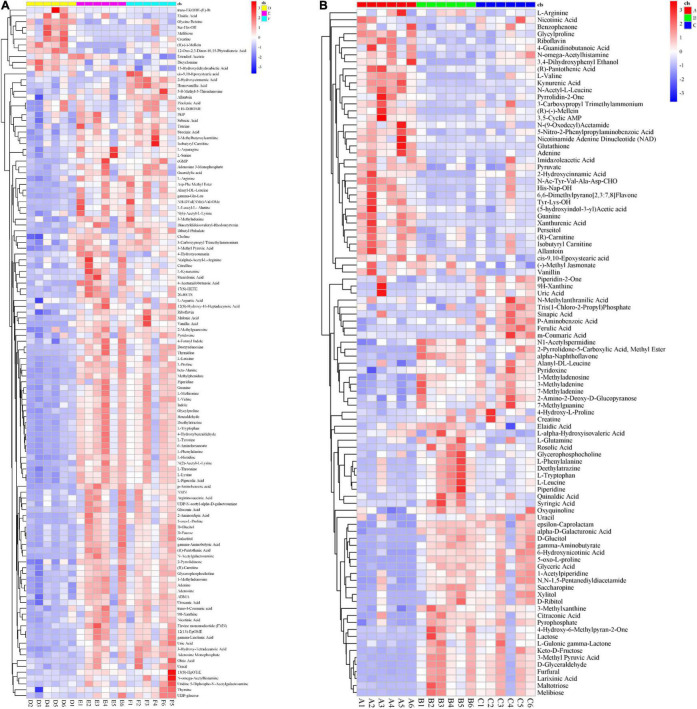
Global impact of *Clpksl8* on secondary metabolism. **(A)** The heat map shows that 98 metabolites have been identified from the extracellular secretion. Red (the left) represents the metabolites are from the WT; Green (the middle) represents the metabolites are from the Δ*Clpks18* strain; Blue (the right) represents the metabolites are from the OE-*Clpksl8* strain. **(B)** The heat map shows that 119 metabolites have been identified from the intracellular metabolites. Yellow (the left) represents the metabolites are from the WT; Pink (the middle) represents the metabolites are from the Δ*Clpksl8* strain; Turquoise (the right) represents the metabolites are from the OE-*Clpksl8* strain.

To compare the secondary metabolism of the WT and mutants, they were cultivated in the Fries 3 medium. The relative expression of *Clpks18* was significantly higher in the OE-*Clpks18* strain than the WT strain in the Fries 3 medium compared with the PDA medium (Figures 1D,G). The relative expression of *Clpks18* in OE-*Clpks18* strain was 3.57 times higher than that of the WT strain, which coincides with the faster mycelial melanization of OE-*Clpks18* strain compared with the WT strain (Figure 1F). At the same time, *Clpks18* was also related to the production of M5HF2C toxin. The expression of the *Clpks18* gene was positively correlated with the M5HF2C toxin production (Figure 4C and Supplementary Figure 3). To further explore the effect of *Clpks18* on other secondary metabolic pathways, the metabolomic analysis of extracellular and intracellular metabolic contents of WT strains, Δ*Clpks18* strains, and OE-*Clpks18* strains was performed; 98 metabolites significantly differed between the WT, Δ*Clpks18*, and OE-*Clpks18* strains (Figure 5A); 119 intracellular metabolites were identified in the WT, Δ*Clpks18*, and OE-*Clpks18* strains (Figure 5B). The enrichment analysis of intracellular metabolites showed that 16 pathways were obviously changed inside the cells (Supplementary Table 2); 7 pathways were found to be changed markedly in the extracellular secretion (Supplementary Table 3), which include the glycerolipid metabolism. We found that glycerin was extracellularly secreted into the medium of δ*Clpks18* and OE-*Clpks18* mutant than the WT (Figure 4B and Supplementary Figure 4). OE-*Clpks18* mutants formed hyper-melanized mycelia and conidia; they might produce more glycerin in the hyphae than the WT strain, which generated higher turgor pressure in the hyphae than WT strains. Since the turgor pressure in the hyphae of OE-*Clpks18* mutants is higher than the threshold value, they should release the glycerin into the extracellular space to reduce the excrescent turgor pressure. The lack of melanin in the cell wall of the Δ*Clpks18* mutants could not maintain the high turgor pressure of cells. In order to reduce the turgor pressure of Δ*Clpks18* mutants, they released more glycerin into the extracellular space to adapt themselves to turgor pressure which was lower than the WT strain. The extracellular and intracellular metabolite analysis showed that a compound identified as (R)-(-)-mellein (Supplementary Figure 5) was highly correlated with the relative expression of the *Clpks18* gene (Tables 2, 3). The (R)-(-)-mellein was not detected in the extracellular secretion of Δ*Clpks18* mutants, while the (R)-(-)-mellein production was restored and secreted into the extracellular environment of the complemented *Clpks18* (Figure 4A). The short-chain peptides such as His-Nap-OH, Tyr-Lys-OH, and N-Ac-Tyr-Val-Ala-Asp-CHO were positively correlated with the expression of *Clpks18* gene extracellularly (Figure 4D and Supplementary Figure 4). In addition, the non-ribosomal peptide synthases (NRPS) were influenced by the *Clpks18* gene. It was supposed that M5HF2C toxin, (R)-(-)-mellein, and short-chain peptides were the candidates for virulence factors.

**TABLE 2:** Top 10 substances in the order of Fold Change by multiple comparison test on extracellular secretion.

	WT vs. Δ *Clpks18*_VIP	Mean_WT	SD_WT	Mean_Δ *Clpks18*	SD_Δ *Clpks18*	Fold change	*p*-value	FDR
(R)-(-)-Mellein	1.39	4,357,616	2,443,826	142,075	15,088	30.67	0.00	0.01
N-Ac-Tyr-Val-Ala-Asp-CHO	1.49	5,016,092	2,705,449	186,917	194,255	26.84	0.00	0.01
Glutathione	1.32	47,425,648	33,494,174	2,771,006	1,646,844	17.12	0.01	0.04
Kynurenic acid	1.60	65,487,645	26,579,480	4,550,339	6,062,841	14.39	0.00	0.00
Tyr-Lys-OH	1.42	27,567,758	16,183,030	2,067,811	2,415,722	13.33	0.00	0.02
2-Hydroxycinnamic acid	1.44	4,418,814	2,065,428	428,074	154,163	10.32	0.00	0.01
His-Nap-OH	1.52	226,127,030	105,875,467	21,969,198	24,796,108	10.29	0.00	0.01
Allantoin	1.32	47,605,591	27,899,392	5,150,391	2,269,200	9.24	0.00	0.02
3-carboxypropyl trimethylammonium	1.44	1568,926,056	825,779,678	229,917,103	122,116,936	6.82	0.00	0.02

	**OE-*Clpks18* vs. Δ*Clpks18*_VIP**	**Mean_OE-*Clpks18***	**SD_OE-*Clpks18***	**Mean_Δ*Clpks18***	**SD_Δ*Clpks18***	**Fold Change**	***p*-value**	**FDR**

Sinapic acid	2.17	1,919,833	79,3784	102,435	4,162	18.74	0.00	0.02
(R)-(-)-Mellein	2.13	2,401,670	1,063,361	142,075	15,088	16.90	0.00	0.02
m-Coumaric acid	2.04	14,852,051	7,520,448	1,055,917	159,991	14.07	0.00	0.04
2-Hydroxycinnamic acid	2.02	3,004,617	1,418,434	428,074	154,163	7.02	0.00	0.04
3,4-Dihydroxyphenyl ethanol	2.12	10,943,280	2,810,528	3,076,021	2,492,454	3.56	0.00	0.03
Uric acid	1.50	5,520,519	3,900,631	1,686,822	331,494	3.27	0.04	0.27
Kynurenic acid	1.97	14,264,016	5,166,754	4,550,339	6,062,841	3.13	0.01	0.32
9H-Xanthine	2.42	10,684,454	3,010,056	4,505,625	436,547	2.37	0.00	0.09
Nicotinic acid	1.81	123,701,250	56,491,845	62,952,954	11,259,224	1.96	0.03	0.41
3,5-Cyclic AMP	1.69	752,359	263,006	414,442	103,138	1.82	0.02	0.17

**TABLE 3:** Top 10 substances in the order of Fold Change by multiple comparison test on intracellular metabolites.

	WT vs. Δ *Clpks18*_VIP	Mean_WT	SD_WT	Mean_Δ *Clpks18*	SD_Δ *Clpks18*	Fold change	*p*-value	FDR
(R)-(-)-Mellein	1.72	3,373,640	1,121,464	185,425	5,950	18.19	0.00	0.00
Ser-His-OH	1.39	2,261,011,817	1,538,157,386	177,076,864	123,599,794	12.77	0.01	0.04
Melibiose	1.19	94,828,462	83,018,414	8,359,615	6,409,352	11.34	0.03	0.09
Glycine betaine	1.46	5,044,618,292	2,177,464,722	1,446,366,720	983,723,002	3.49	0.00	0.03
Creatine	1.19	89,141,346	57,738,346	25,857,899	18,133,344	3.45	0.03	0.09
Dicyclomine	1.47	77,082,871	7,394,681	58,482,891	9,805,145	1.32	0.00	0.03
12-Oxo-2,3-Dinor-10,15-Phytodienoic acid	1.57	3,055,430	174,070	2,677,602	93,334	1.14	0.00	0.01

	**OE-Clpks18 vs. Δ*Clpks18*_VIP**	**Mean_OE-*Clpks18***	**SD_OE-*Clpks18***	**Mean_Δ*Clpks18***	**SD_Δ*Clpks18***	**Fold change**	***p*-value**	**FDR**

(R)-(-)-Mellein	2.34	2,087,784	608,940	185,425	5,950	11.26	0.00	0.01
2-Hydroxycinnamic acid	2.25	20,989,628	4,617,226	7,100,966	3,108,265	2.96	0.00	0.02
cis-9,10-Epoxystearic acid	1.73	17,488,636	8,815226	6,192,771	3,004,008	2.82	0.01	0.19
Glycine betaine	1.69	3254,280,581	1,481,103,965	1,446,366,720	983,723,002	2.25	0.03	0.41
Homovanillic acid	2.06	20,272,674	4,962,582	9,480,956	3,281,891	2.14	0.00	0.07
9,10-DiHOME	1.51	394,895,243	145,578,924	207,924,102	129,879,481	1.90	0.04	0.30
Pinolenic acid	1.63	801,815,554	298,787,954	441,518,658	227,696,267	1.82	0.04	0.45
Dibutyl phthalate	1.66	109,020,376	23,779,796	66,554,880	35,613,748	1.64	0.04	0.42
Allantoin	1.80	183,955,689	31,513,853	119,051,071	38,350,076	1.55	0.01	0.16
12-Oxo-2,3-Dinor-10,15-Phytodienoic Acid	1.91	2,879,035	97,364	2,677,602	93,334	1.08	0.00	0.13

### The Contribution of (R)-(-)-Mellein to the Virulence of *Curvularia lunata*

**FIGURE 6:**
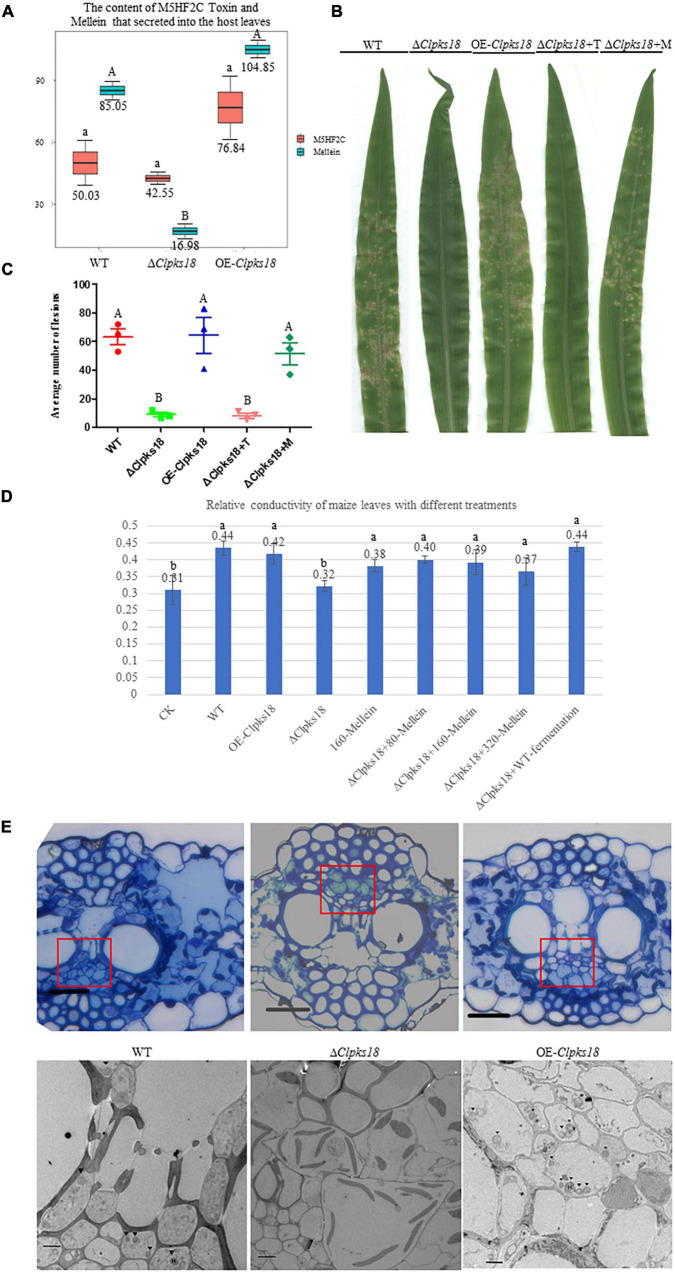
(R)-(-)-Mellein is a vital factor for the virulence of *C. lunata*. **(A)** The content of M5HF2C Toxin and (R)(-)-Mellein that secreted into the host leaves. The M5HF2C toxin from maize leaves infected by WT and mutants at 72 hpi was extracted and quantified by UPLC-MS. Data are presented as the average concentration in maize leaves and the bars represent the median ± maximum and minimum. Content unit (ng/g). The different capital letters indicate statistical significance (*p* < 0.01) and the different lowercases indicate statistical significance (*p* < 0.05) using Tukey’s multiple comparison test. **(B)** The bioassay of mutants and WT in maize. The leaves of ZHENGDAN-958 at five-leaf stage was inoculated with a 0.05% Tween-20 suspension of I × 10^6^ conidia per ml for 7 days. Δ*Clpksl8*+T means Δ*Clpksl8* conidia suspension with 60 ng/100 μl M5HF2C toxin; Δ*Clpksl8*+M means Δ*Clpksl8* conidia suspension with 80 ng/100 μl (R)-(-)-Mellein. **(C)** Dot plot showing the average number of disease lesions observed in each individual leaf harvested (*n* = 3). The different capital letters indicate statistical significance (*p* < 0.01) using Tukey’s multiple comparison test. **(D)** Effects of different treatments on the membrane injury of the maize leaves at 24 hpi compared with *C. lunata* inoculation and Blank control (*n* = 3). Δ*Clpksl8*+M means Δ*Clpksl8* conidia suspension with 80–320 ng/100 μl (R)(-)-Mellein. Data are means ± Standard Deviation (SD). Different letters denote significant differences based on Tukey’s Multiple Comparison Test (*P* < 0.05). **(E)** The observation in the cells of maize leaves’ phloem at 120 hpi. The scale bars represent 25 μm in pictures of optical microscope; red panes show the position for transmission electron microscope (TEM) observation. The scale bars represent 4 μm in the TEM picture of Δ*1Clpksl8*. The scale bars represent 2 μm TEM picture of the WT and OE-*Clpksl8*. ▼ show the position of the hyphae.

To confirm the key virulence factor among the two, namely, M5HF2C toxin and (R)-(-)-mellein, they were detected in the maize leaves infected by the WT, Δ*Clpks18*, and OE-*Clpks18* strains at 3 dpi (72 hpi). It was found that the M5HF2C toxin (50.03 ng/g) and (R)-(-)-mellein (85.05 ng/g) were secreted into the host cells (Figure 6A and Supplementary Figures 6, 7). To verify which of (R)-(-)-mellein and M5HF2C is the more important pathogenic factor for *C. lunata*, these two compounds were used for the virulence assays. When Δ*Clpks18* conidial suspension is inoculated into maize leaves with (R)-(-)-mellein at a concentration of 800 ng/ml, the pathogenicity of Δ*Clpks18* mutant is largely restored to the level of WT. However, as Δ*Clpks18* conidial suspension is inoculated into maize leaves with M5HF2C toxin at a concentration of 600 ng/ml, the pathogenicity of Δ*Clpks18* mutant could not be restored to the level of WT (Figures 6B,C). At 24 hpi., the maize leaves infected by the WT, Δ*Clpks18*, OE-*Clpks18*, and Δ*Clpks18* strains with different concentrations of M5HF2C toxin and (R)-(-)-mellein and fermentation of WT in liquor were collected for the relative conductivity test. The relative conductivity test indicated the membrane injury of the maize leaves. It showed that the Δ*Clpks18* in the early stages of infection could not cause obvious injury on the membrane of maize leaves cells. The leaves infected by WT, OE-*Clpks18*, and Δ*Clpks18* with different concentrations of (R)-(-)-mellein and fermentation of WT in liquor showed significant differences with blank control. At the same time, the single treatment with 160 ng/ml (R)-(-)-mellein can cause the relative conductivity to increase when compared with the blank control. The relative conductivity does not increase with the increase in the treatment concentration of (R)-(-)-mellein. The results revealed that (R)-(-)-mellein plays a key role in the early stages of *C. lunata* infection. Only as the Δ*Clpks18* conidia combine infection with the fermentation of WT in liquor, could the level of damage to the host cells’ membrane was restored to the level of WT infection. It implied that the fermentation of WT in liquor contains some substances that play a role in *C. lunata* infection except (R)-(-)-mellein (Figure 6D). Also, it suggests that the (R)-(-)-mellein secretion was correlated with the expression of *Clpks18* gene. High hyphal content was observed in the phloem of maize leaves infected with WT and OE-*Clpks18* strains at 5 days (at 120 hpi) using the transmission electron microscope. However, hyphal content in the phloem of maize leaves infected with the Δ*Clpks18* was very lower than the WT (Figure 6E).

## Discussion

The *Clpks18* gene plays an important role in the pathogenicity of *C. lunata*. It synthesizes 1,3,6,8-tetrahydroxynaphthalene (Supplementary Figure 8), which is essential for the synthesis of DHN-melanin in both mycelia and conidia. According to the previous research, a differentiated cell wall rich in DHN-melanin was found to be essential for the generation of turgor to maintain the rigid structure to prevent the efflux of solutes in *Magnaporthe oryzae* (Chumley and Valent, 1990). The appressorium generated the turgor up to 8.0 MPa by accumulating high concentrations of glycerol and other polyols. Too high concentration of glycerol would collapse appressoria. The test results showed that the concentration of 1.75 M glycerol collapsed 52% of appressoria (de Jong et al., 1997). The translation of appressorium turgor into mechanical force causes a narrow penetration of hyphae to emerge from the base of the appressorium and breach the cuticle of the rice leaf (Talbot, 2003). The DHN-melanin and turgor are required for the appressorium-mediated infection of *M. oryzae*. The deletion of turgor pressure sensor gene *sln1* produced the excess intracellular appressorium turgor pressure and hyper-melanized non-functional appressoria and does not organize the septins and form the penetration peg that is required for leaf infection (Ryder et al., 2019). Similarly, in this study, the OE-*Clpks18* mutant generated higher turgor pressure in Fries 3 medium (Figure 4B). It revealed that OE-*Clpks18* strain might generate higher turgor pressure in conidia and hyphae than the WT strain during the infection process. However, the excess level of higher turgor pressure in conidia and hyphae does not lead to stronger virulence. The formation of appressoria was observed during the *C. lunata* infection. The DHN-melanin was not accumulated in the appressoria’s cell wall. It was accumulated in the hyphae and conidia. The hyphae of WT, Δ*Clpks18*, and OE-*Clpks18* strains can penetrate the cellophane of the same stiffness regardless of whether they generate appressoria or not. The hyphae invading the host through either stomata or penetrating directly into the cuticle on the leaf surface were witnessed (Supplementary Figure 9). While *S. turcica* invaded the maize at 10-leaf stage, the appressoria produced were higher at the 4-leaf stage. At the same time, the production of DHN-melanin was positively correlated with the hardness of the medium the *S. turcica* grows in Liu et al. (2020). It is supposed that appressoria might play a role during the infection, especially as *C. lunata* infect the leaves during the older stage, which contain the stronger barriers. Therefore, it suggests that melanin might be one of the accessory factors for the infection. In addition, some other important factors are also required for the pathogenicity of *C. lunata*. In fact, the *Clpks18* impact many pathway that related to secondary metabolism (Supplementary Tables 1, 2). Some secondary metabolism such as Arginine biosynthesis metabolism are related to the pathogenicity of the pathogen (Zhang et al., 2015).

The secretion of short peptides by the WT into the external environment might play an important role in the host infection (Figure 5B). A linear, C-terminally reduced and D-amino acid residue-rich octapeptide named fusaoctaxin A of *Fusarium graminearum* was synthesized by the 2 NPRSs. Fusaoctaxin A is a virulence factor required for the cell-to-cell invasion of wheat and interferes with plasmodesmata callose accumulation to prevent plasmodesmata closure upon fungal infection. Fusaoctaxin A has been identified only in pathogenic *F. graminearum*, but not in the non-pathogenic *Fusarium.* The deletion of cluster *fg3_54* essential for fusaoctaxin A biosynthesis reduced the pathogenicity against wheat. The microscopic observation showed that the hyphae of the *fg3_54* deleted mutant could not penetrate into the host cell. The addition of fusaoctaxin A into the *fg3_54* deleted mutant and non-pathogenic *Fusarium* restored the pathogenicity of *Fusarium* (Jia et al., 2019). The short-chain peptides secreted by the WT strain and OE-*Clpks18* strain have some structural similarities with the fusaoctaxin A; therefore, they might help the pathogen to colonize in the host cells during the infection process. Further research is required to know the mechanism of short peptides of *C. lunata* during infection.

M5HF2C toxin was identified as a non-host-specific toxin and causes lesions on the leaves of monocotyledon plants, such as *Z. mays, O. sativa*, and *S. bicolor*, and dicotyledon plants, such as *C. annuum* (Liu et al., 2009). The content of M5HF2C of each mutant in the Fries 3 medium was consistent with the expression of *Clpks18* gene. In the Fries 3 medium, the *Clpks18* deleted mutants’ acetaldehyde dehydrogenase low expression (unpublished data), which maybe related to the M5HF2C biosynthesis (Peng et al., 2019). However, the difference between the content of M5HF2C toxin in maize leaves infected by WT and other mutants was not significant (Figure 6A). The Δ*Clpks18* conidial suspension with M5HF2C toxin at a concentration of 600 ng/ml does not recover the virulence of *C. lunata* (Figure 6B). As described, the maize ZHENGDAN-958 was a resistance variety. So, the results suggest that the M5HF2C toxin might be an accessory factor for the pathogenicity of *C. lunata*, or maybe it needs other cooperative factors to help when *C. lunata* infect the maize of resistance variety.

The metabolomics results showed that the concentration of (R)-(-)-mellein was positively correlated with *Clpks18* gene expression in the Fries 3 medium. The secretion of (R)-(-)-mellein in the maize leaves infected with WT strain and other mutants was similar to that of the secretion in the Fries 3 medium. Adetunji et al. (2018) reported that (R)-(-)-mellein produced from *Lasiodiplodia pseudotheobromae* has cytotoxicity against several monocotyledon weeds. Adetunji et al. (2018) confirmed the cytotoxicity of (R)-(-)-mellein in dicotyledon-Valerianaceae. Previous research has shown that *C. lunata* can colonize in many plants’ leaves to cause leaf spots. (R)-(-)-mellein of *Parastagonospora nodorum* was synthesized by the partial reduction of PKS (PR-PKS), SNOG_00477 (SN477); 200 μg/ml of (R)-(-)-mellein inhibited the germination of wheat (*Triticum aestivum*) and *Medicago truncatula* seeds. However, *P. nodorum* Δ*sn477* mutants did not show any significant differences on the virulence against wheat compared with the WT (Chooi et al., 2015). *P. nodorum* is a necrotrophic fungus, in which various host-selective toxins-necrotrophic effectors infect the host tissue during colonization. It causes blotch on wheat leaves and glumes, which lead to shriveling of wheat kernels. This study showed that (R)-(-)-mellein was secreted into maize leaves during the infestation process of *C. lunata*. The concentration of (R)-(-)-mellein is more than 5 times higher in the leaves infected with WT compared with the Δ*Clpks18* mutants. Furthermore, the leaves infected with OE-*Clpks18* strain also produced a higher concentration of (R)-(-)-mellein compared with the leaves infected with WT strain (Figure 6A). With the 800 ng/ml (R)-(-)-mellein, the virulence of the Δ*Clpks18* strain has almost been recovered to the WT strain (Figure 6B). However, the OE-*Clpks18* strain is not more virulent than the WT strain (Figure 6C). At 24 hpi, with the increase in the (R)-(-)-mellein concentration (800–3,200 ng/ml), the virulence does not increase obviously. The concentration of the (R)-(-)-mellein from 800 to 3,200 ng/ml can cause slight injury to the maize leaves (Figure 6D). At the 7th day, the maize leaves infected by Δ*Clpks18* conidial suspension with 800 ng/ml (R)-(-)-mellein showed lesion spots like those with the WT and OE-*Clpks18* strains. As we observed the phloem of the maize leaves infected by WT, OE-*Clpks18*, and Δ*Clpks18* strains, large numbers of hyphae were observed in the leaves infected by WT and OE-*Clpks18* strains. In Δ*Clpks18* strains infected leaves, the hyphae were almost unseen. It implies that (R)-(-)-mellein is an important pathogenic factor for *C. lunata* in the earlier stage of the infection.

## Conclusion

In conclusion, the virulence factors such as (R)-(-)-melanin, short-chain peptide, and M5HF2C toxin might play a role in the *C. lunata* infection. Compared with the M5HF2C toxin, (R)-(-)-mellein is a vital factor for the pathogenicity of *C. lunata* in the earlier stage of the infection. Nevertheless, *Clpks18* gene attributes to the pathogenicity of *C. lunata* by impacting the complex metabolic network. During the whole infection process, there may be more virulence factors to be explored.

## Data Availability Statement

The data presented in the study are deposited in the Metabolights repository, accession number MTBLS4244.

## Author Contributions

JC conceived the project. ZL completed the experiments and wrote the manuscript. SW and KD assisted with the experiments. JR helped to analyze the metabolome. All authors contributed to the article and approved the submitted version.

## Conflict of Interest

JR was employed by the Suzhou PANOMIX Biomedical Tech Co., Ltd. The remaining authors declare that the research was conducted in the absence of any commercial or financial relationships that could be construed as a potential conflict of interest.

## Publisher’s Note

All claims expressed in this article are solely those of the authors and do not necessarily represent those of their affiliated organizations, or those of the publisher, the editors and the reviewers. Any product that may be evaluated in this article, or claim that may be made by its manufacturer, is not guaranteed or endorsed by the publisher.
