# Supplementary material for: The Interpretation of the Role of a Polyketide Synthase ClPKS18 in the Pathogenicity of Curvularia lunata
Source: Front Microbiol. 2022 May 24;13:853140. doi: 10.3389/fmicb.2022.853140 (PMC9171202; doi:10.3389/fmicb.2022.853140)
Supplement: Supplementary file 1 [file Data_Sheet_1.pdf]

Supplementary

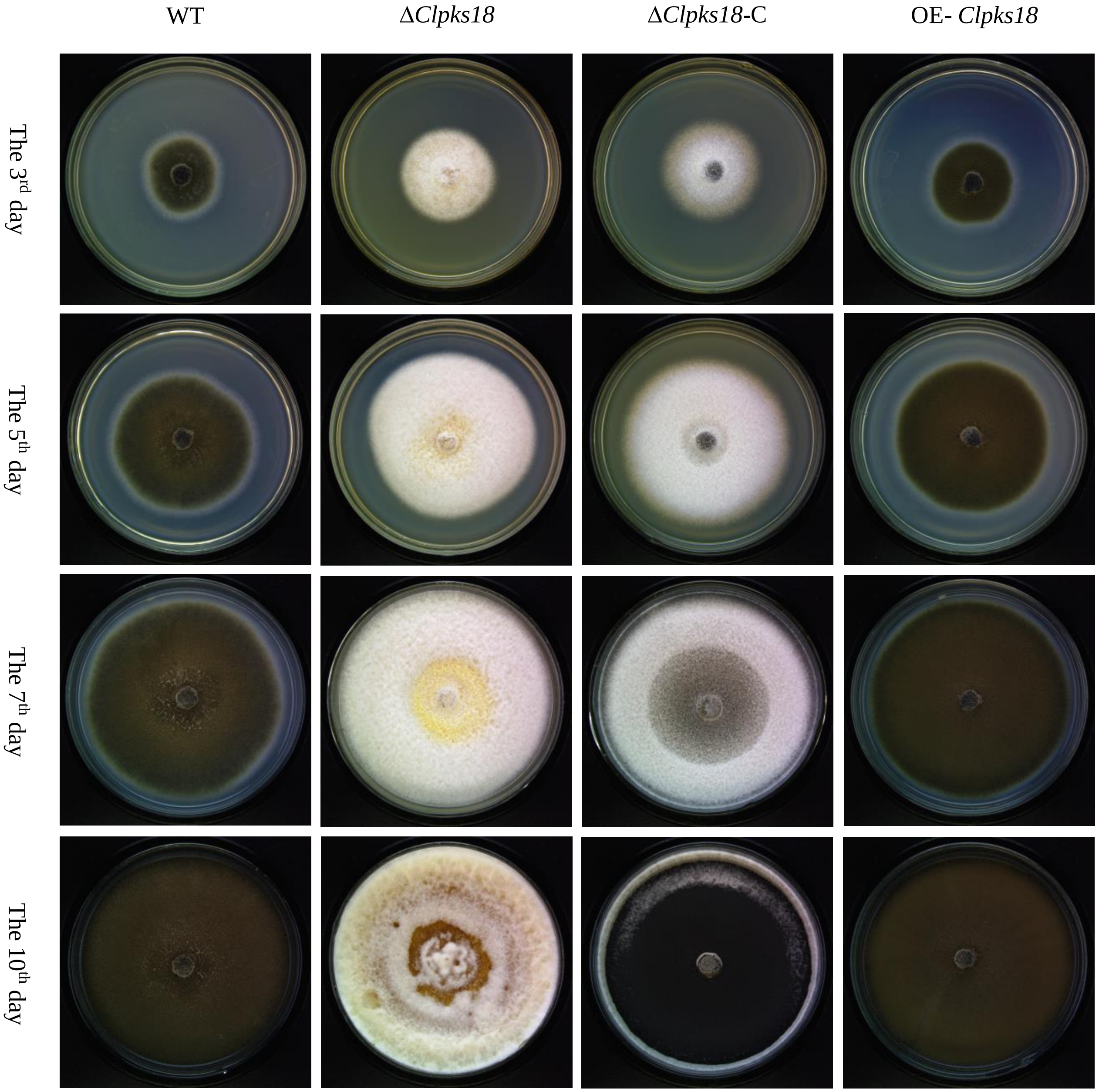

FIGURE 1. Cultures of *Curvularia lunata* WT, the Δ*Clpks18* mutant , *Clpks18*-C strain and OE-*Clpks18* mutant grown on PDA medium from the 3rd day to 10th day at 28 °C.

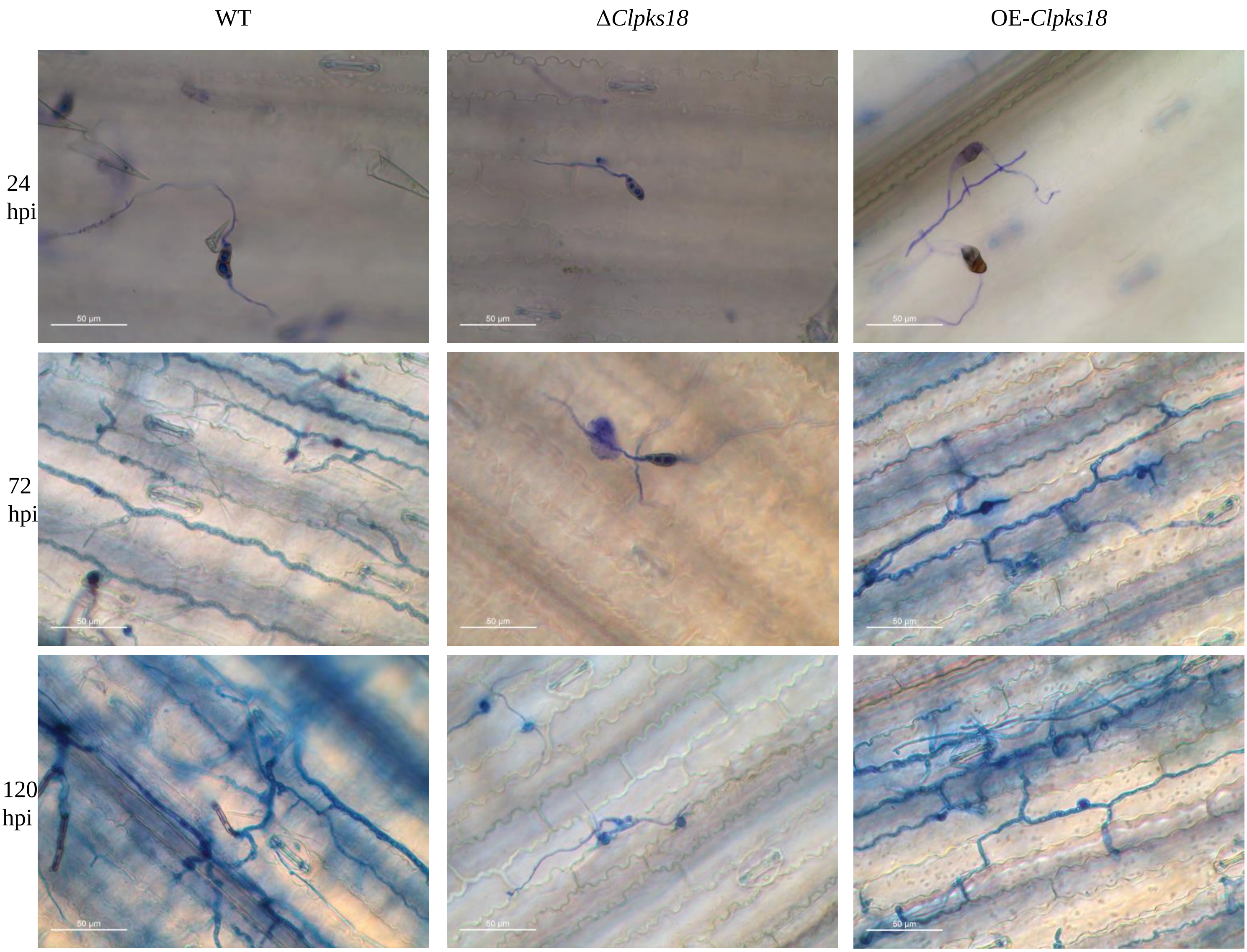

FIGURE 2. The infection process of the WT strain, the Δ*Clpks18* mutant and OE-*Clpks18* mutant of *C. lunata*.

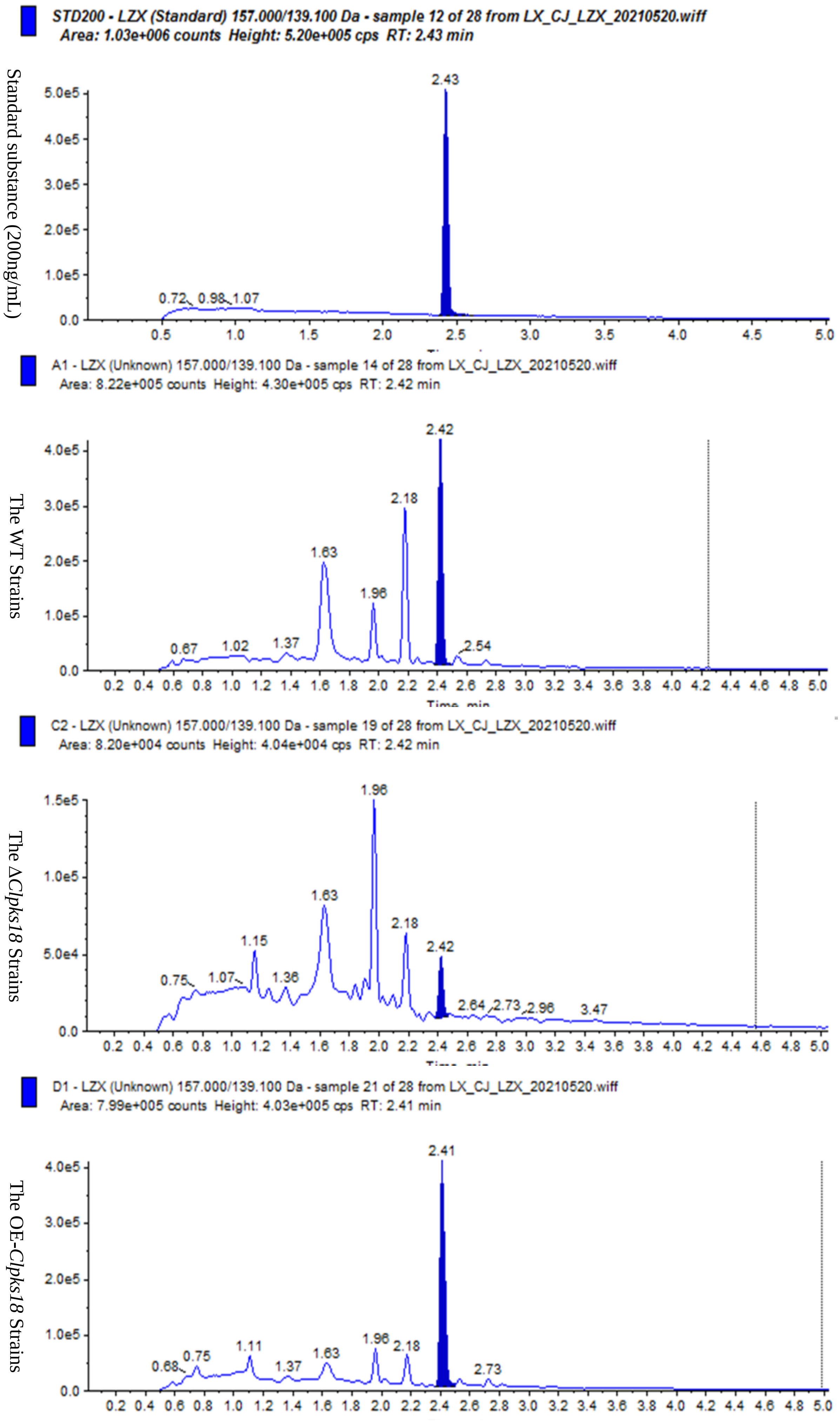

FIGURE 3. M5HF2C toxin content detection by HPLC-MS in the Fries 3 medium

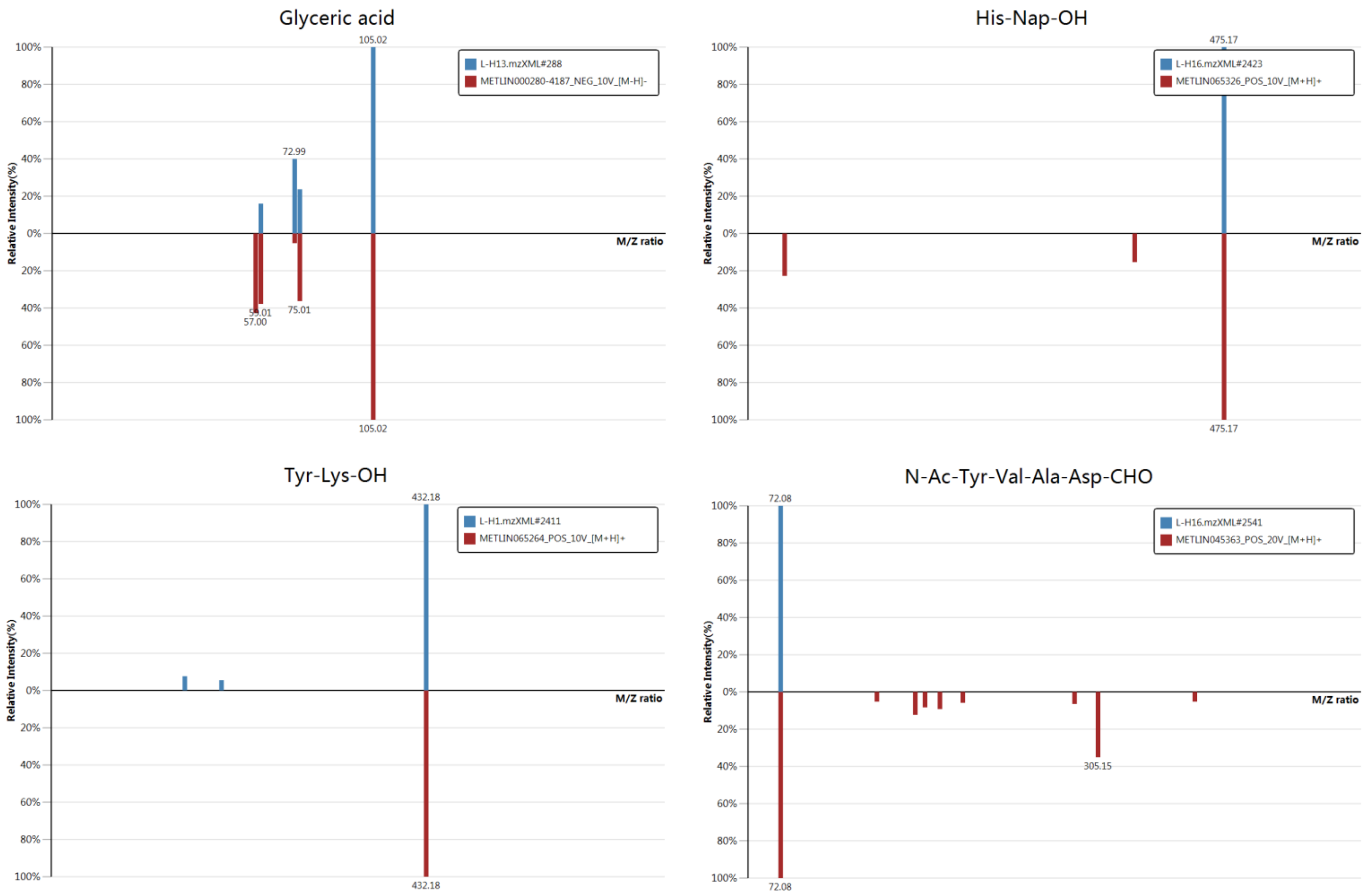

FIGURE 4. The substances identification by Mass spectrometry database comparison

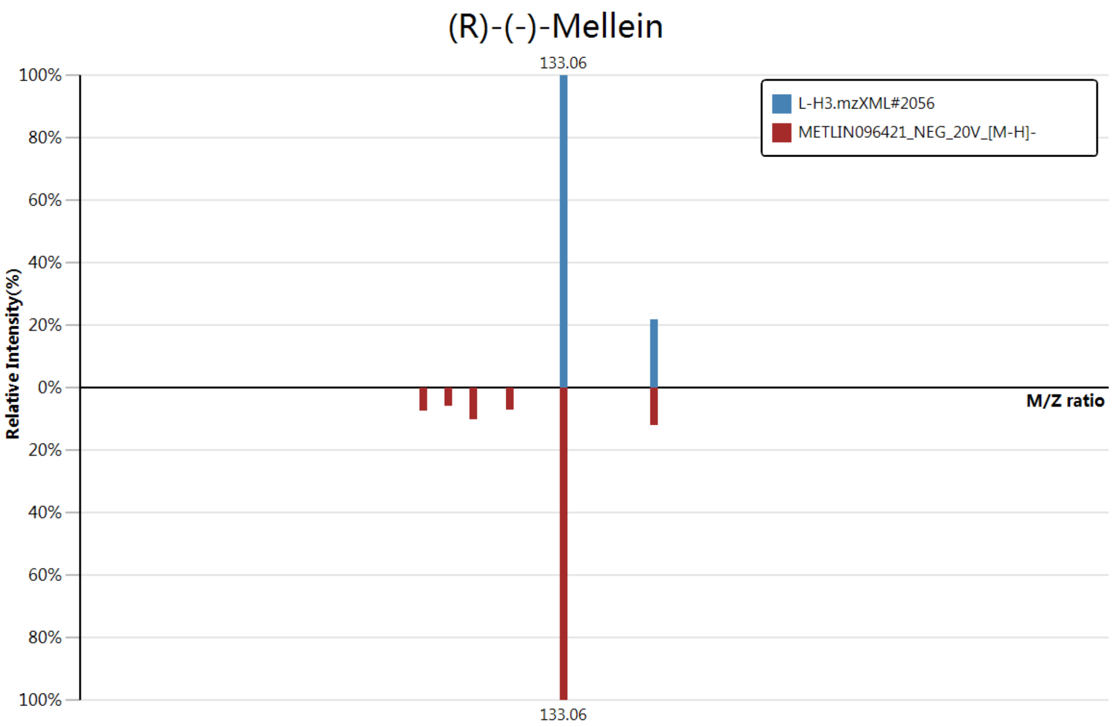

FIGURE 5. (R)-(-)-Mellein identification by Mass spectrometry database comparison

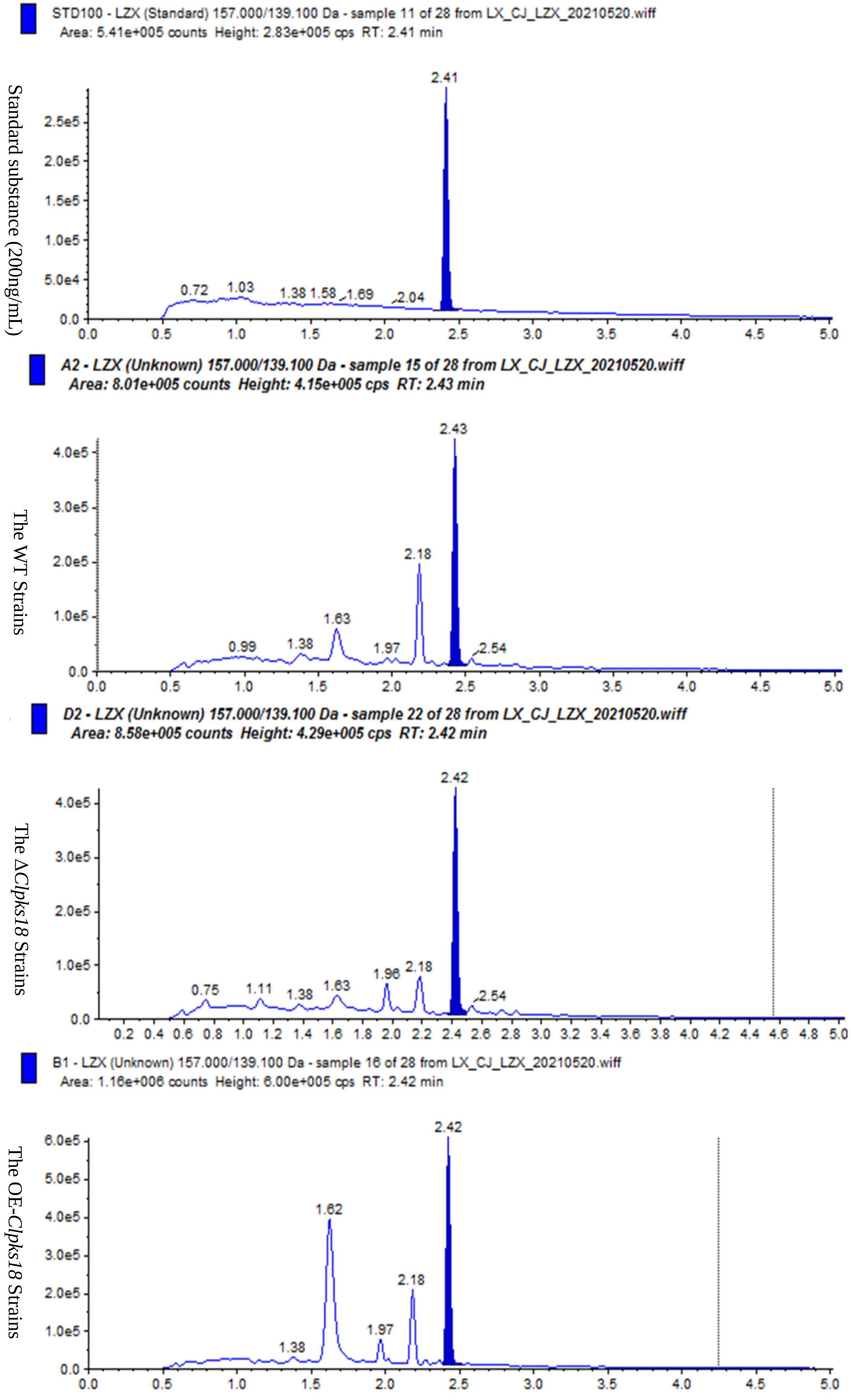

FIGURE 6. M5HF2C toxin content detection by HPLC-MS in the maize leaves infected by *C. lunata* and its mutants

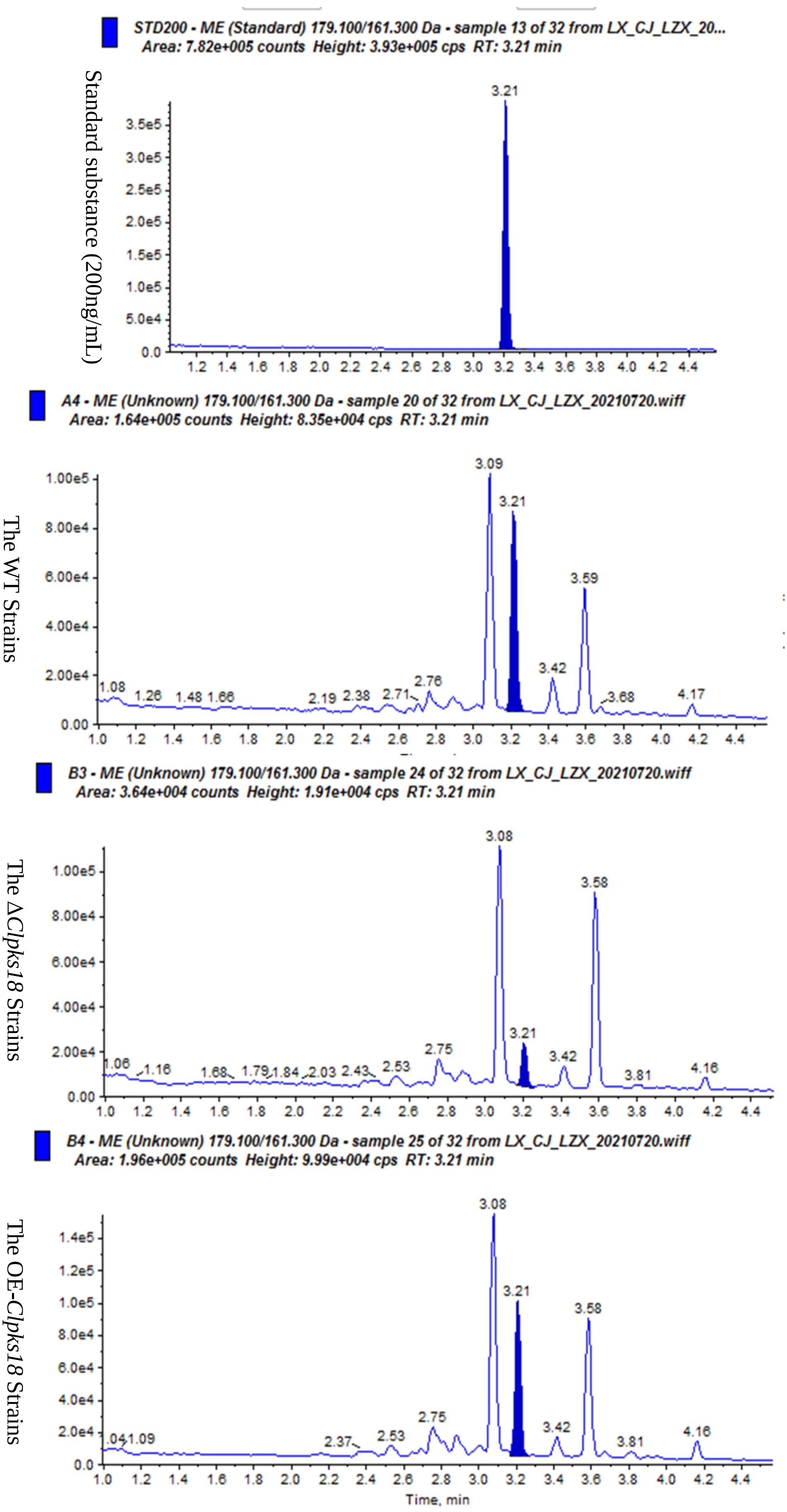

FIGURE 7. (R)-(-)-mullein content detection by HPLC-MS in the maize leaves infected by *C. lunata* and its mutants

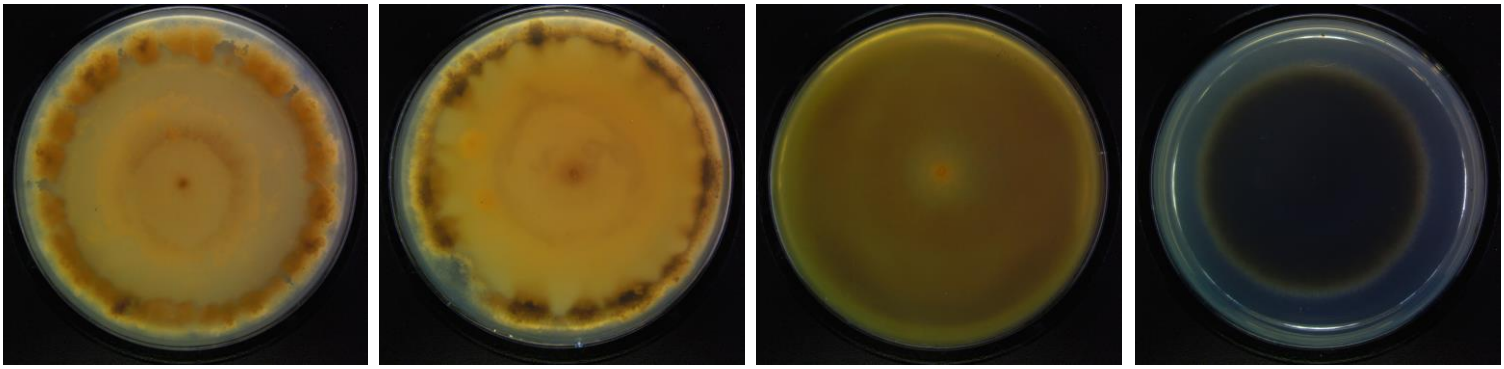

Δ*Clpks18*+20mg1,3,6,8-THN Δ*Clpks18*+100mg1,3,6,8-THN Δ*Clpks18*+200mg1,3,6,8-THN Δ*Clpks18*+1000mg1,3,6,8-THN Reverse

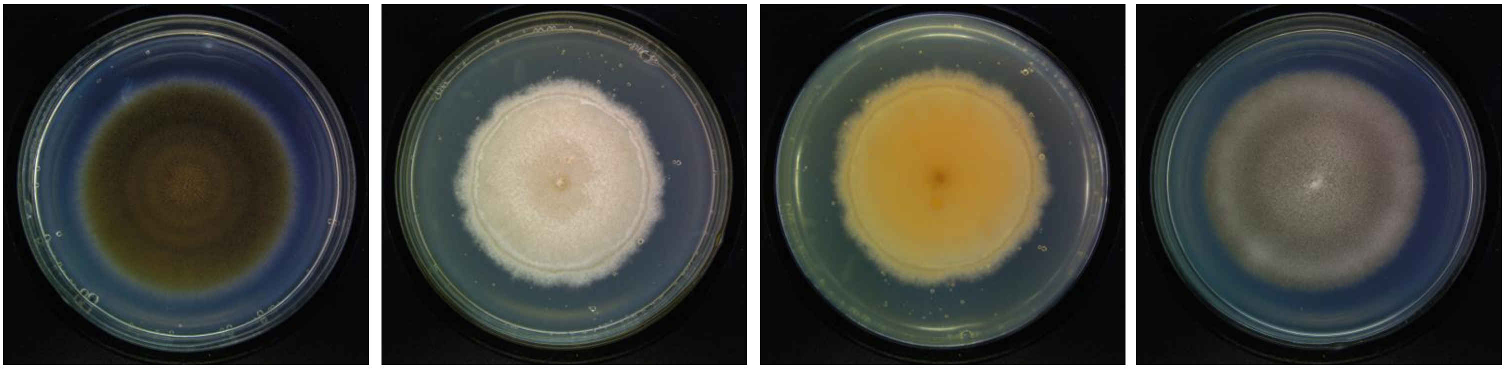

WT+10mg DMSO Δ*Clpks18*+10mL DMSO Frontage Δ*Clpks18*+10mL DMSO Reverse Δ*Clpks18*+1000mg1,3,6,8-THN Frontage

FIGURE 8. Replenishment Experiment of 1,3,6,8-tetrahydroxynaphthalene (1,3,6,8-THN); the 1,3,6,8-THN solution is prepared with stock solution (100mg 1,3,6,8-THN dissolves in 1mL Dimethyl sulfoxide (DMSO).

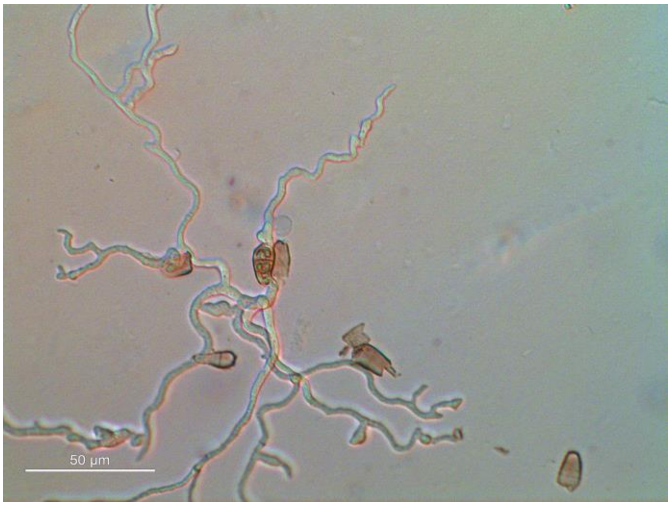

The conidia of WT strain on the semipermeable membrane at 12 hpi

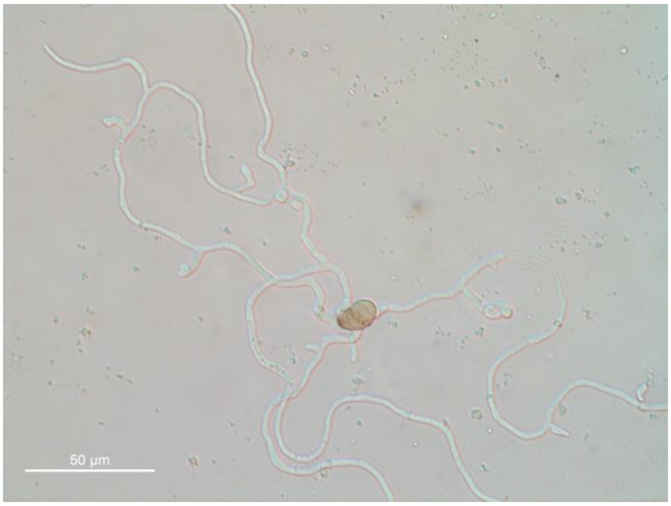

The conidia of OE-*Clpks18* mutant on the semipermeable membrane at 12 hpi

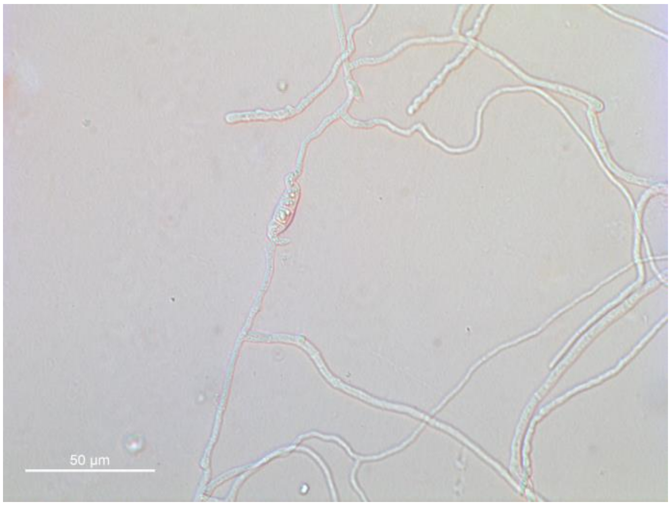

The conidia of Δ*Clpks18* mutant on the semipermeable membrane at 12 hpi

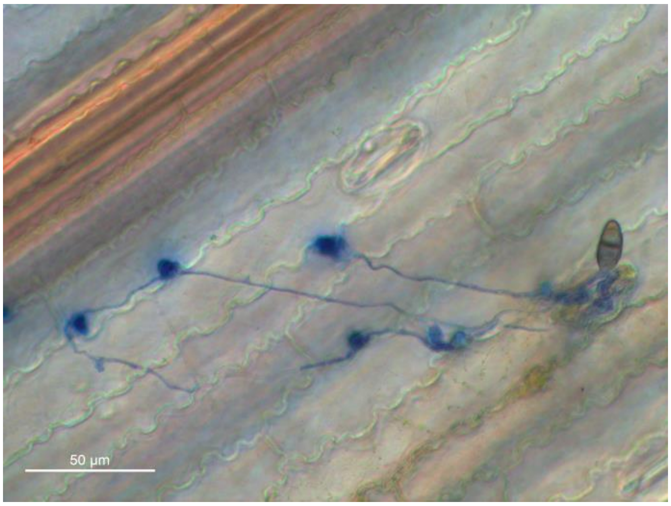

The conidia of WT strain on the semipermeable membrane at 48 hpi

FIGURE 9. The conidia of WT strain and mutants on the semipermeable membrane and leaf surface. As we observed, the semipermeable membrane was penetrated by the conidia during about 48-72 hpi.

Table1. Primers for gene complementation and over-expression and Real-time quantitative PCR

Primer names	Sequences (5’ to 3')）
QF1	ATGTGTTATTAAGTTGGATCC GGAGGTCAACACATCAATGCC
QR1	GATGATGACATCCAT TTGGATGCTTGGGTAGAATAGGTAAG
PKS18a1	TCACCATTGCGCAATTGTCGC
PKS18R1-F	AGAAACTCGAGCTACT ATAAAATTCCAACGCCAACATCTCT
FF1	AGTAGCTCGAGTTTCTCCATAATAATGTGT
FR1	GATGTGTTGACCTCCGAATTC AACTTAATAACACATTGCGGACGT
QF1-1	CAGGTCGACTCTAGAGGATCC GGAGGTCAACACATCAATGCCTATT
PKS18R1-F1	TATGGAGAAACTCGAG ATAAAATTCCAACGCCAACATCTCT
FF1-1	CTCGAGTTTCTCCATAATAATGTGTGAGTAGT
FR1-1	GATGTGTTGACCTCCGAATTC AACTTAATAACACATTGCGGACGTTT
PKS18a2	TGTTTGATGGGTTGGGCAGCTCT

QF1/QR1 for *TrpC* promoter amplification and infusion with *Clpks18* gene and vector;
PKS18a-1/PKS18R1-F for *Clpks18* gene amplification and infusion with *TrpC* promoter and terminator;
FF1/FR1 for *TrpC* terminator amplification and infusion with *Clpks18* gene and vector;
QF1-1/PKS18R1-F1 for *C. lunata* promoter and *Clpks18* gene amplification and infusion with *TrpC* terminator and vector;
FF1-1/FR1-1 for *TrpC* terminator amplification and infusion with *Clpks18* gene and vector;
PKS18a1/ PKS18a2 for Real-time quantitative PCR detecting the *Clpks18* gene expression.

Table2. Impacts of *Clpks18* on intracellular metabolites

	Total	Hits	Raw p	-log(p)	Holm adjust	FDR	Impact
Riboflavin metabolism	20	2	0.05	2.94	1.00	0.34	0.44
beta-Alanine metabolism	25	5	0.00	9.43	0.01	0.00	0.39
Arginine and proline metabolism	68	4	0.04	3.28	1.00	0.32	0.26
Pantothenate and CoA biosynthesis	25	4	0.00	6.84	0.16	0.02	0.23
Histidine metabolism	33	2	0.13	2.07	1.00	0.62	0.21
Tropane; piperidine and pyridine alkaloid biosynthesis	25	3	0.01	4.53	1.00	0.14	0.20
Purine metabolism	80	10	0.00	13.31	0.00	0.00	0.19
Pyrimidine metabolism	67	5	0.01	4.84	1.00	0.12	0.18
Lysine degradation	44	3	0.05	3.03	1.00	0.33	0.18
Lysine biosynthesis	34	2	0.13	2.02	1.00	0.63	0.17
alpha-Linolenic acid metabolism	27	1	0.40	0.91	1.00	1.00	0.17
Tryptophan metabolism	53	2	0.26	1.34	1.00	0.95	0.16
Cysteine and methionine metabolism	57	3	0.09	2.41	1.00	0.53	0.16
Nicotinate and nicotinamide metabolism	50	4	0.01	4.29	1.00	0.14	0.12
Glycine; serine and threonine metabolism	47	5	0.00	6.39	0.24	0.03	0.11
Arginine biosynthesis	23	1	0.35	1.04	1.00	1.00	0.11

Multivariate statistical analysis used in this analysis included: Principal Component Analysis (PCA), Partial Least Squares-Discriminant Analysis (PLS-DA) and Orthogonal Partial Least Squares Discriminant Analysis (OPLS-DA).
The impact ≥ 10% was showed in the table

Table 3. Impacts of *ClPKS18* on extracellular secretion

	Total	Hits	Raw p	-log(p)	Holm adjust	FDR	Impact
Glutathione metabolism	37	2	0.08	2.54	1.00	1.00	0.27
Riboflavin metabolism	20	1	0.23	1.49	1.00	1.00	0.20
Pantothenate and CoA biosynthesis	25	3	0.00	5.62	0.55	0.27	0.15
Furfural degradation	10	1	0.12	2.12	1.00	1.00	0.14
Nicotinate and nicotinamide metabolism	50	3	0.02	3.70	1.00	0.79	0.13
Glycerolipid metabolism	28	2	0.05	3.03	1.00	1.00	0.12
Pyrimidine metabolism	67	1	0.58	0.55	1.00	1.00	0.11

Multivariate statistical analysis used in this analysis included: Principal Component Analysis (PCA), Partial Least Squares-Discriminant Analysis (PLS-DA) and Orthogonal Partial Least Squares Discriminant Analysis (OPLS-DA). The impact $\geq$ 10% was showed in the table.

Table 4. Fries 3 media

	quality
yeast extract	1g
sucrose	20g
KH_2PO_4	1g
$MgSO_4 \cdot 7H_2O$	0.5g
NH_4NO_3	1g
$CaCl_2 \cdot 2H_2O$	0.13g
$C_4H_{12}N_2O_6$	5g

Dissolve in 1L of water, adjust pH to 3, $C_4H_{12}N_2O_6$ is ammonium tartrate.
